# Advocating *"spine damage control" *as a safe and effective treatment modality for unstable thoracolumbar fractures in polytrauma patients: a hypothesis

**DOI:** 10.1186/1752-2897-3-6

**Published:** 2009-05-11

**Authors:** Philip F Stahel, Michael A Flierl, Ernest E Moore, Wade R Smith, Kathryn M Beauchamp, Anthony Dwyer

**Affiliations:** 1 Department of Orthopaedic Surgery, Denver Health Medical Center, University of Colorado School of Medicine, 777 Bannock Street, Denver, Colorado 80204, USA; 2 Department of Surgery, Denver Health Medical Center, University of Colorado School of Medicine, 777 Bannock Street, Denver, Colorado 80204, USA; 3 Division of Neurosurgery, Denver Health Medical Center, University of Colorado School of Medicine, 777 Bannock Street, Denver, Colorado 80204, USA

## Abstract

**Background:**

The "ideal" timing and modality of fracture fixation for unstable thoracolumbar spine fractures in multiply injured patients remains controversial. The concept of "damage control orthopedics" (DCO), which has evolved globally in the past decade, provides a safe guidance for temporary external fixation of long bone or pelvic fractures in multisystem trauma. In contrast, "damage control" concepts for unstable spine injuries have not been widely implemented, and the scarce literature in the field remains largely anecdotal. The current practice standards are reflected by two distinct positions, either (1) immediate "early total care" or (2) delayed spine fixation after recovery from associated injuries. Both concepts have inherent risks which may contribute to adverse outcome.

**Presentation of hypothesis:**

We hypothesize that the concept of "spine damage control" – consisting of immediate posterior fracture reduction and instrumentation, followed by scheduled 360° completion fusion during a physiological "time-window of opportunity" – will be associated with less complications and improved outcomes of polytrauma patients with unstable thoracolumbar fractures, compared to conventional treatment strategies.

**Testing of hypothesis:**

We propose a prospective multicenter trial on a large cohort of multiply injured patients with an associated unstable thoracolumbar fracture. Patients will be assigned to one of three distinct study arms: (1) Immediate definitive (anterior and/or posterior) fracture fixation within 24 hours of admission; (2) Delayed definitive (anterior and/or posterior) fracture fixation at > 3 days after admission; (3) "Spine damage control" procedure by posterior reduction and instrumentation within 24 hours of admission, followed by anterior 360° completion fusion at > 3 days after admission, if indicated. The primary and secondary endpoints include length of ventilator-free days, length of ICU and hospital stay, mortality, incidence of complications, neurological status and functional recovery.

**Implications of hypothesis:**

A "spine damage control" protocol may save lives and improve outcomes in severely injured patients with associated spine injuries.

## Background

Polytrauma patients have a severely deranged immune response, characterized by an early excessive activation of innate immunity (hyperinflammation), followed by a delayed attenuation of adaptive immunity with decreased T-cell function (immunosuppression) and enhanced susceptibility to infection, sepsis, and multiple organ failure (MOF) [1,2]. In addition, about a third of all multiply injured patients have severe disturbances of their clotting system on arrival to the emergency department, as determined by the presence of postinjury coagulopathy [3,4]. This subset of patients has an increased incidence of MOF and death compared to severely injured patients who are not coagulopathic [3-6]. Historically, a "damage control" protocol was first advocated in terms of an abbreviated laparotomy for patients "in extremis" with massive bleeding from abdominal trauma, in order to allow restoration from the "lethal triad" of coagulopathy, hypothermia, and persistent metabolic acidosis [7,8]. Aside from the traumatic "1^st ^hit", the pathophysiological disturbance of the immune and clotting systems render multiply injured patients prone to sustaining aggravating "2^nd ^hit" injuries related to inadequate timing and modality of surgical procedures [9,10]. This notion has been well recognized for the management of long bone and pelvic fractures in polytrauma, leading to a worldwide implementation of the "damage control orthopedics" (DCO) strategy in the past decade [11]. The concept of DCO entails a staged management of long bone and pelvic fractures by acute external fixation and delayed (scheduled) conversion to internal fixation during a physiological "time-window of opportunity" [1,9,10].

While the concepts and timing of fracture fixation for isolated spinal injuries – with or without neurological compromise – are well defined in the pertinent literature, the question about the "ideal" time-point of spine fracture fixation in severely injured patients remains an ongoing topic of debate. Few studies have assessed the impact of timing of spine fracture fixation on non-neurological outcome and complications. In a landmark article, Croce and colleagues performed a retrospective analysis of a prospective database on 291 consecutive patients with unstable spine fractures requiring surgical fixation [12]. Patients were matched for injury severity and stratified by level of spine injury into two distinct cohorts, depending on the timing of fracture fixation: "early" fixation (within 3 days, *n *= 142) versus "late" fixation (> 3 days, *n *= 149). The authors found that the early fixation of thoracic spine fractures resulted in a lower incidence of pneumonia, fewer ventilator-dependent days, a shorter ICU stay, and reduced hospital charges [12]. More recently, Cengiz *et al*. reported data from a randomized prospective pilot study on 27 patients who underwent surgical stabilization of an unstable fracture in the thoracolumbar region from T8 to L2 [13]. Patients were allocated to two different groups based on the timing of surgery of definitive spine fracture fixation, either within 8 hours (*n *= 12) or more than 3 days (*n *= 15). The authors found that those patients who underwent spine fixation within 8 hours had a significantly decreased incidence of pulmonary complications, such as pneumonia, and shorter length of ICU and hospital stay, compared to the group with delayed spine fixation [13]. Although this study has some significant shortcomings, such as the small patient population and the flawed randomization procedure based on the individual surgeon's operative schedule, these preliminary findings make a strong point that early spine fracture fixation is feasible and safe, and potentially beneficial for multiply injured patients [13]. This notion was confirmed by a recent systematic review of the pertinent peer-reviewed literature in the field [14]. In their review, Rutges *et al*. analyzed all published articles in Medline and Embase databases which provided a comparison between different time-points of surgical stabilization of thoracic or lumbar spine fractures [14]. Ten papers encompassing 1,427 patients met the inclusion criteria. Based on their systematic review, the authors concluded that the early intervention for fracture stabilization in the thoracolumbar spine is safe, advantageous, and associated with a significantly decreased incidence of postoperative complications [14].

However, due to the lack of high level scientific evidence from prospective randomized trials, a consensus on the "ideal" timing of spine fracture fixation in multisystem trauma has not yet been reached. Advocates of early spine fixation cite multiple intuitive advantages when managing severely injured patients with unstable spine fractures. Prolonged bed rest and the inability of adequate positioning and mobilization of polytrauma patients have been associated with severe posttraumatic complications. These include the development of pressure sores, pulmonary complications, and thromboembolic events. Multiply injured patients are at additional increased risk of sustaining such adverse events secondary to their profound immunological dysfunction, as outlined above [1,2]. Polytrauma patients require unrestricted options of mobilization and positioning in the ICU, including the upright seated position for treatment of head injuries and prone positioning for respiratory therapy of pulmonary complications, such as the acute respiratory distress syndrome (ARDS) [15]. Last but not least, any unfixated thoracolumbar fracture may contribute to the "antigenic load" of trauma by increasing stress and pain, which will add up to the overall trauma burden to the organism and turn the physiological "host defense response" into a pathological "host defense failure disease" [1,2]. This rationale provides a strong argument for the early clearance of bed rest and log-roll precautions in multiply injured patients and forms the basis of a "spine damage control" concept in severely injured patients [9,16,17].

## The hypothesis

We hypothesize that the concept of "spine damage control" will provide a safe and effective treatment modality for unstable thoracolumbar fractures in multiply injured patients, associated with less complications and improved outcomes compared to conventional treatment strategies. We define "spine damage control" as a staged procedure of immediate posterior fracture reduction and instrumentation within 24 hours ("day 1 surgery") [9,17], followed by scheduled 360° completion fusion during a physiological "time-window of opportunity" (> 3 days after trauma), if an adjunctive anterior decompression and fusion is indicated for neurological or biomechanical reasons. This concept differs from the more common elective strategy of a staged spine fixation by initial posterior fixation and delayed anterior completion by its timeliness (posterior fixation within 24 h) and expanded applicability to all unstable thoracolumbar fractures, including pure anterior column burst fractures (AO/OTA type A3).

**Figure 1:**
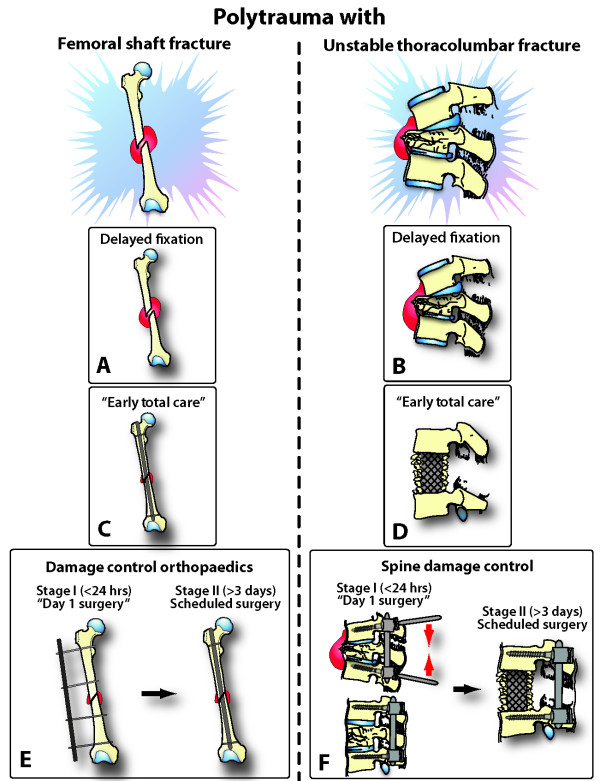
**Analogy of management strategies for femur shaft fractures versus unstable thoracolumbar spine fractures in multiply injured patients**. See text for details and explanations.

The rationale for "spine damage control" is illustrated in analogy to the management of femur shaft fractures in polytrauma patients, by extrapolating three main therapeutic options to the situation of an unstable thoracolumbar fracture: (1) "Early total care" with definitive fixation on day 1; (2) Initial non-operative management with delayed fixation; (3) "Damage control" procedure on day 1, with scheduled conversion to definitive fixation at a later time-point (Figure 1). There is a wide consensus on the notion that femur shaft fractures should be stabilized as early as possible in polytrauma patients, in order to avoid potentially lethal complications [18,19]. The option of initial non-operative management and delayed stabilization of femur fractures is considered antiquated and obsolete in modern polytrauma management strategies (Figure 1A). The 2^nd ^therapeutic option of "early total care" for femur shaft fracture fixation (Figure 1C) has been widely abandoned due to increased morbidity and mortality of multiply injured patients, in favor of a DCO strategy characterized by initial external fixation and scheduled conversion to intramedullary nail fixation (Figure 1E) [11,20-23].

**Figure 2:**
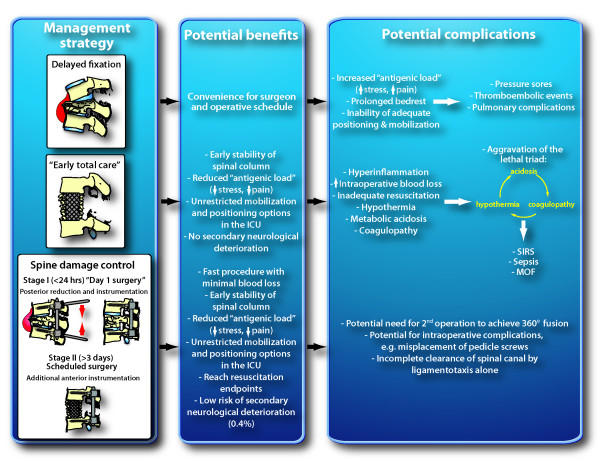
**Risk/benefit assessment of three distinct management strategies for unstable thoracolumbar fractures in polytrauma patients**. See text for details and explanations. ICU, intensive care unit; MOF, multiple organ failure; SIRS, systemic inflammatory response syndrome.

In contrast, "damage control" spine stabilization concepts in multisystem trauma have been addressed scarcely in the peer-reviewed literature and are based on limited anecdotal reviews or case reports [16,17,24]. The currently disseminated practice standards for fixation of unstable thoracolumbar fractures in polytrauma patients consist of either (1) a passive approach of delayed spine fixation after recovery from associated injuries (Figure 1B), or (2) the aggressive concept of immediate "early total care" by invasive anterior approaches, vertebral corpectomy, decompression, and anterior fusion (Figure 1D). The concept of "no touch" in the early management of unstable thoracolumbar spine fractures (Figure 1B) is based on the notion that multiply injured patients are "too sick" to be operated on within the first few days after major trauma. Another common rationale for postponing acute spine fixation is based on inconvenience of timing or unavailability of surgical capabilities [13]. Similarly, the approach of "early total care" for unstable spine fractures is frequently based on the ideal "time-window of convenience" for the surgeon, e.g. by having operative room capabilities available by chance [13]. Both concepts – conservative caution and aggressive primary fixation – bear inherent risks and dangers for severely injured patients (Figure 2). On one hand, keeping patients on prolonged bed rest and log-roll precautions will increase the risk of complications and prevent adequate mobilization and positioning in the ICU. On the other hand, the immediate full anterior decompression and fusion is associated with extensive anterior approaches, prolonged operative times, aggravated hypothermia and increased blood loss, particularly in presence of postinjury coagulopathy [25]. In addition, bleeding sources from cancellous bone (corpectomy) and epidural venus plexus (spinal canal decompression) will aggravate the "vicious cycle" of traumatic hemorrhage and coagulopathy (Figure 2) [26]. This notion is supported by data from the recent literature, demonstrating a significantly increased mortality from 2.5% to 7.6% by early definitive spine fixation within 48 hours after trauma [27]. Interestingly, an encompassing prospective multicenter study published in the German literature by the spine working group of the German Trauma Society ("Arbeitsgemeinschaft Wirbelsaeule der Deutschen Gesellschaft fuer Unfallchirurgie") revealed that 65.7% of all polytrauma patients with operative thoracolumbar fractures (*n *= 682) are currently treated by exclusive posterior fixation in German and Austrian level 1 trauma centers [28]. The authors showed that posterior instrumentation was associated with a decreased complication rate of 4.7%, compared to 10.8% in the primary anterior fusion group [28]. Impressively, only 0.4% of all patients with posterior instrumentation showed a secondary neurological deterioration, implying that the "spine damage control" procedure is safe from a neurological perspective (Figure 2).

**Figure 3:**
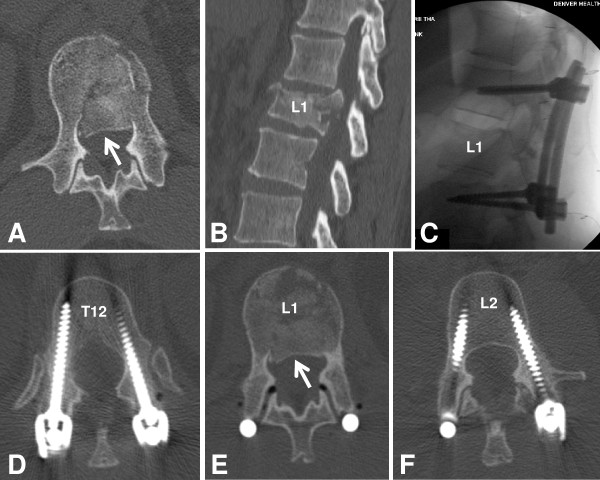
**Clinical example of "spine damage control" for an unstable L1 complete burst fracture (AO/OTA 53-A3.3) with 50% spinal canal narrowing (A, B) in a 50-year old lady who sustained an axial loading trauma mechanism in a commercial airliner crash at Denver International Airport**. The patient was neurologically intact (ASIA grade E). She was taken for a posterior reduction and two-level instrumentation T12-L2 on day 1. Intraoperative fluoroscopy films show an excellent reduction of the L1 burst fracture with restoration of near-anatomic vertebral body height and sagittal profile in lordosis (C). The postoperative CT scan (D**-**F) demonstrates a significant clearance of the anterior spinal canal fragment by pure ligamentotaxis (arrow in E, compared to arrow in A). The patient tolerated the operative procedure well. She was mobilized on postoperative day (POD) #1 with physical therapy and discharged on POD #2 to fly back to her hometown, where she followed up with a local spine surgeon for anterior completion fusion. This example emphasizes the safety and efficacy of the "spine damage control" concept.

Preliminary data from our own institution emphasize the safety and efficacy of the "spine damage control" concept for unstable thoracolumbar fractures in polytrauma patients. We detected a significant reduction of pneumonia, and a complete eradication of pressure sores, in 21 consecutive polytrauma patients with unstable thoracolumbar fractures, after formal implementation of the "spine damage control" modality as a mandatory clinical care standard. The injured levels in this preliminary cohort with a mean ISS of 54.5 points included T2/T3, T3/T4, T4/T5, T5/T6 (n = 3), T8/T9 (n = 2), T10/T11 (n = 3), T12/L1 (n = 6), L2 (n = 2), L3, L4. None of the patients died as a result of their injuries, or as a consequence of an overly aggressive spine fixation strategy (Stahel *et al*., unpublished observations). Thus, our preliminary data suggest that "spine damage control" represents a save and efficient modality for staged spine fixation in this selected cohort of multiply injured patients (Figure 3).

Based on the rationale that both concepts of delayed fixation and primary "early total care" spinal fusion are considered harmful and associated with adverse outcome (Figure 2), we propose to test the concept of "spine damage control" (Figure 1F) as a potentially safe and efficient modality of spine fixation in multiply injured patients with unstable thoracolumbar fractures.

## Testing the hypothesis

A prospective multicenter trial on a large cohort of multiply injured patients may be able to clarify the optimal timing of fixation for unstable thoracolumbar fractures, comparing the following distinct treatment modalities:

(1) Immediate definitive (anterior and/or posterior) fracture fixation within 24 hours of admission

(2) Delayed definitive (anterior and/or posterior) fracture fixation at > 3 days after admission;

(3) "Spine damage control" procedure by posterior reduction and instrumentation within 24 hours of admission, with a staged anterior completion fusion at > 3 days, if indicated.

For selected patients, the posterior "spine damage control" fixation may be considered the definitive treatment, e.g. in case of a pure osseous flexion/distraction type injury ("Chance" fracture, AO/OTA type B2.1). All other patients may benefit from an adjunctive 2^nd ^intervention for anterior 360° completion fusion, as determined by biomechanical (anterior column stabilization) and/or neurological (anterior spinal canal decompression) indications. The 2^nd ^procedure should be performed at > 3 days, in order to bridge the acute phase of hyperinflammation and to ensure adequate resuscitation from traumatic hemorrhage and coagulopathy, in order to reduce the intraoperative risk of bleeding from cancellous bone and epidural veins [9].

Performing a randomized prospective trial on this topic does not appear ethically feasible. This is based on the consideration that "spine damage control" is a safe strategy for multiply injured patients and that the allocation to a "delayed care" or "early total care" treatment arm will put the patient's safety at risk. In fact, we have implemented the "spine damage control" practice as a mandatory clinical care standard at Denver Health Medical Center in 2008. This protocol mandates the clearance from log-roll precautions in any patient with a thoracolumbar fracture within 24 hours of admission, either by clearance and mobilization of patients with stable fracture patterns, or by early posterior reduction and instrumentation on day 1. Preliminary prospective data from our institution show a significant reduction of pressure sores and pulmonary complications after implementation of this clinical care standard, suggesting that "spine damage control" is feasible and safe for the management of polytrauma patients with unstable thoracolumbar fractures (Stahel *et al*., unpublished observations).

### Inclusion criteria

Age ≥ 18 years, Injury Severity Score (ISS) > 15 points, presence of unstable thoracolumbar fracture (AO/OTA location 52- or 53-) with or without spinal cord injury (ASIA scores A-E).

### Exclusion criteria

Age < 18 years, stable thoracolumbar fracture amenable to non-operative treatment, patients *"in extremis" *with an unjustifiable risk of a severe complication or death related to a spine surgical procedure performed on day 1.

### Primary outcome parameter

Length of ventilator-free days/ICU days.

### Secondary outcome parameters

Mortality; rate of complications, including ARDS, MOF, thromboembolic events, pressure sores, infections; long-term clinical outcome. Patients will be followed clinically for up to 1 year after trauma and assessed for radiographic outcome (fusion, maintenance of fixation), neurological impairment (ASIA score), and functional outcome (Oswestry disability index, spine VAS score). The hypothesis may be confirmed by demonstrating a lower incidence of mortality and complications, and an improved long-term outcome in the "spine damage control" group, compared to the two conventional study groups.

## Implications of the hypothesis

In summary, there is currently no consensus on the "ideal" timing and modality of spine fracture fixation in multiply injured patients. Delaying surgical fixation of unstable spine fractures in multisystem trauma has been associated with an increased risk of severe complications attributed to restrictions in mobilization by maintaining prolonged log-roll precautions. On the other hand, early spine fixation within the first 48 hours after trauma ("early total care") has been associated with a significantly increased mortality [27], which is likely due to exacerbation of the "lethal triad" due to increased blood loss in hypothermic and coagulopathic trauma patients [5,9,25]. Based on these insights, the new concept of "spine damage control" should be advocated as a safe staged procedure of early posterior reduction and instrumentation, followed by a scheduled anterior completion during the physiological "time-window of opportunity". Although this concept appears supported by common sense from a patient safety perspective, a scientific proof of concept is currently still lacking. The safety and feasibility of the "spine damage control" strategy will have to be validated in a well-designed, prospective multicenter trial on a large cohort of multiply injured patients. The long-term dissemination and implementation of "spine damage control" will likely lead to a significant reduction of posttraumatic morbidity and mortality in these critically ill patients.

## Competing interests

The authors declare that they have no competing interests.

## Authors' contributions

PFS wrote the first version of the manuscript. MAF designed the graphic artwork in figures 1 &2. All authors provided modifications to the text and approved the final version of the manuscript.
